# Genomes of Bacteriophages Belonging to the Orders *Caudovirales* and *Petitvirales* Identified in Fecal Samples from Pacific Flying Fox (*Pteropus tonganus*) from the Kingdom of Tonga

**DOI:** 10.1128/mra.00038-22

**Published:** 2022-02-17

**Authors:** Jasmine K. M. Lopez, Maketalena Aleamotu’a, Viliami Kami, Daisy Stainton, Michael C. Lund, Simona Kraberger, Arvind Varsani

**Affiliations:** a Biodesign Center for Fundamental and Applied Microbiomics, School of Life Sciences, Center for Evolution and Medicine, Arizona State University, Tempe, Arizona, USA; b School of Environmental and Life Sciences, The University of Newcastle, Callaghan, New South Wales, Australia; c Land Resource Division, The Pacific Community, Narere Campus, Suva, Fiji; d Department of Entomology and Plant Pathology, Division of Agriculture, University of Arkansas System, Fayetteville, Arkansas, USA; e Structural Biology Research Unit, Department of Integrative Biomedical Sciences, University of Cape Town, Cape Town, South Africa; DOE Joint Genome Institute

## Abstract

Twenty-nine circular genomes of bacteriophages in the orders *Caudovirales* and *Petitvirales* were identified from fecal samples from Pacific flying foxes that were collected from their roosting sites on the Pacific Island of Tonga in 2014 and 2015. The vast majority are microviruses (*n* = 25), with 2 siphoviruses, 1 myovirus, and 1 podovirus.

## ANNOUNCEMENT

Pacific flying foxes (*Pteropus tonganus*) are frugivorous bats that are found throughout the Pacific region (1) and are the sole bat species found on the Tongan archipelago (2), playing an important role in pollination and seed dispersal (1, 3, 4). Our previous work on Pacific flying foxes from Tonga reported various cressdnaviruses (5). Here, we expand on that work, focusing on bacteriophages.

Fecal samples from four roosting sites (‘Atele, Ha'avakatolo, Kolovai, and Lapaha) located on Tongatapu island were collected in April 2014 and January 2015 (5). From each, 5- to 10-g samples were pooled based on sample year, resuspended in 45 mL of SM buffer (50 mM Tris HCl, 10 mM MgSO_4_, 0.1 M NaCl [pH 7.5]), and processed for viral nucleic acid extraction as described by Male et al. (5). The High Pure viral nucleic acid kit (Roche Diagnostics, USA) was used to extract viral DNA. The extracted DNA samples were enriched for circular sequences using rolling circle amplification with the TempliPhi 100 kit (GE Healthcare, USA); they were then used by Beijing Genomics Institute (Hong Kong) to prepare 2 × 90-bp libraries using their custom protocol, and the libraries were sequenced using a HiSeq 2000 sequencer (Illumina, USA). The raw reads were trimmed with Trimmomatic v0.39 (6) and then *de novo* assembled using metaSPAdes v3.12.0 (7). In order to identify bacteriophage-like sequences, we used VirSorter (8), and sequences were determined to be circular (based on terminal redundancy). From the pooled samples from April 2014 (Tbat1) and from January 2015 (Tbat2), 29 full bacteriophage genomes were identified. Open reading frames were identified with RASTtk (9), and the annotations were refined with the HMMER web server with the Pfam database (10) and Cenote-Taker 2 (11). The coverage depth and number of mapped reads for each genome were determined with BBMap (12). All bioinformatic software was used with default settings. The genomes have varied read depths of 10.99× to 24,465.65×, with 741 to 1,592,983 reads (Table 1).

**TABLE 1:** Summary of bacteriophage genomes determined from Tongan bat fecal samples and their top BLASTn hit information

Family	GenBank accession no.	Length (nt)	GC content (%)	Read depth (×)	No. of mapped reads	Data for top BLASTn hit				
						GenBank accession no.	Virus type^*a*^	Query coverage (%)	E value	Identity (%)
*Microviridae*	OL617014	4,315	36.80	21.1775	1,019	MH617689	*Microviridae* sp. isolate ctbe975	17	1.00E−49	68.61
*Microviridae*	OL617015	4,179	55.20	26.3508	1,228	MH992220	Apis mellifera-associated microvirus 41 INH_SP_302	15	2.00E−48	69.41
*Microviridae*	OL617016	6,049	54.40	150.1405	10,117	MT310373	*Microvirus* sp. isolate 1712115_248	56	0	68.49
*Microviridae*	OL617017	6,376	54.80	53.9305	3,828	MN988476	*Rhizobium* phage RHph_TM1_7A	51	0	72.17
*Microviridae*	OL617018	6,260	55.30	133.1529	9,282	MT310373	*Microvirus* sp. isolate 1712115_248	60	0	68.05
*Microviridae*	OL617019	6,153	53.90	93.6808	6,414	MT310373	*Microvirus* sp. isolate 1712115_248	59	0	68.24
*Microviridae*	OL617020	6,111	59.40	422.1753	28,704	MK765588	Tortoise microvirus 38 isolate 38_SP_76	53	0	70.08
*Microviridae*	OL617021	6,096	55.40	10.9934	747	MK765589	Tortoise microvirus 39 isolate 39_SP_82	18	2.00E−69	69.44
*Microviridae*	OL617022	6,062	57.50	41.7634	2,820	MK765589	Tortoise microvirus 39 isolate 39_SP_82	20	2.00E−76	71.33
*Microviridae*	OL617023	5,964	38.10	38.0241	2,525	BK033080	TPA: *Microviridae* sp. isolate ctr3K1	86	0	86.82
*Microviridae*	OL617024	5,949	55.50	8,094.895	536,306	MT310362	*Microvirus* sp. isolate 1712115_301	69	0	70.69
*Microviridae*	OL617025	5,857	58.00	24,465.65	1,592,983	MH616837	*Microviridae* sp. isolate ctcf_4	36	3.00E−111	71.96
*Microviridae*	OL617026	5,849	56.40	29.7458	1,940	MT310362	*Microvirus* sp. isolate 1712115_301	73	0	69.02
*Microviridae*	OL617027	5,767	56.80	34.6105	2,228	MK765635	Tortoise microvirus 82 isolate 82_SP_77	28	2.00E−86	70.32
*Microviridae*	OL617028	5,648	40.70	11.9102	749	MH616763	*Microviridae* sp. isolate ctbb565	46	1.00E−133	67.60
*Microviridae*	OL617029	4,969	56.60	25.4431	1,409	BK015202	TPA: *Microviridae* sp. ct2OM3	30	6.00E−144	68.48
*Microviridae*	OL617030	4,580	55.40	16.5537	845	MZ364279	Robinz microvirus RP_139	37	3.00E−135	68.06
*Microviridae*	OL617031	4,440	47.60	31.5509	1,566	MH992217	Apis mellifera-associated microvirus 42 INH_SP_292	34	3.00E−102	66.49
*Microviridae*	OL617032	4,405	44.40	22.8904	1,123	KM589510	*Microviridae* Fen7786_21	2	2.00E−17	77.69
*Microviridae*	OL617033	4,389	47.70	58.9834	2,870	MT309935	*Microvirus* sp. isolate BS1_501	12	2.00E−11	64.20
*Microviridae*	OL617034	4,380	37.50	15.1317	741	MH649004	*Microviridae* sp. isolate ctbd002	11	1.00E−32	71.60
*Microviridae*	OL617035	4,362	48.30	28.903	1,404	MN582079	*Microviridae* sp. ct0DW36	31	8.00E−85	69.94
*Microviridae*	OL617036	4,325	44.30	16.5563	800	MT309934	*Microvirus* sp. isolate BS1_502	14	9.00E−46	67.53
*Microviridae*	OL617037	4,275	60.60	276.2742	13,132	MT310281	*Microvirus* sp. isolate 1712115_698	21	8.00E−104	69.70
*Microviridae*	OL617038	3,911	41.40	19.495	851	MT310293	*Microvirus* sp. isolate 1712115_653	10	1.00E−30	73.93
*Myoviridae*	OL617039	31,988	35.60	4,741.802	1,517,943	MN855801	*Bacteriophage* sp. isolate 103	4	0	82.23
*Podoviridae*	OL617040	51,802	41.70	290.3623	161,065	MN840487	Proteus phage 2207-N35	60	0	74.50
*Siphoviridae*	OL617041	48,082	47.70	388.254	203,214	BK017157	TPA: *Siphoviridae* sp. isolate ctEwT2	70	0	94.03
*Siphoviridae*	OL617042	50,667	48.50	22.8497	12,658	BK057309	TPA: *Siphoviridae* sp. isolate ctWpt2	24	0	72.15

a TPA, third party annotation.

**FIG 1:**
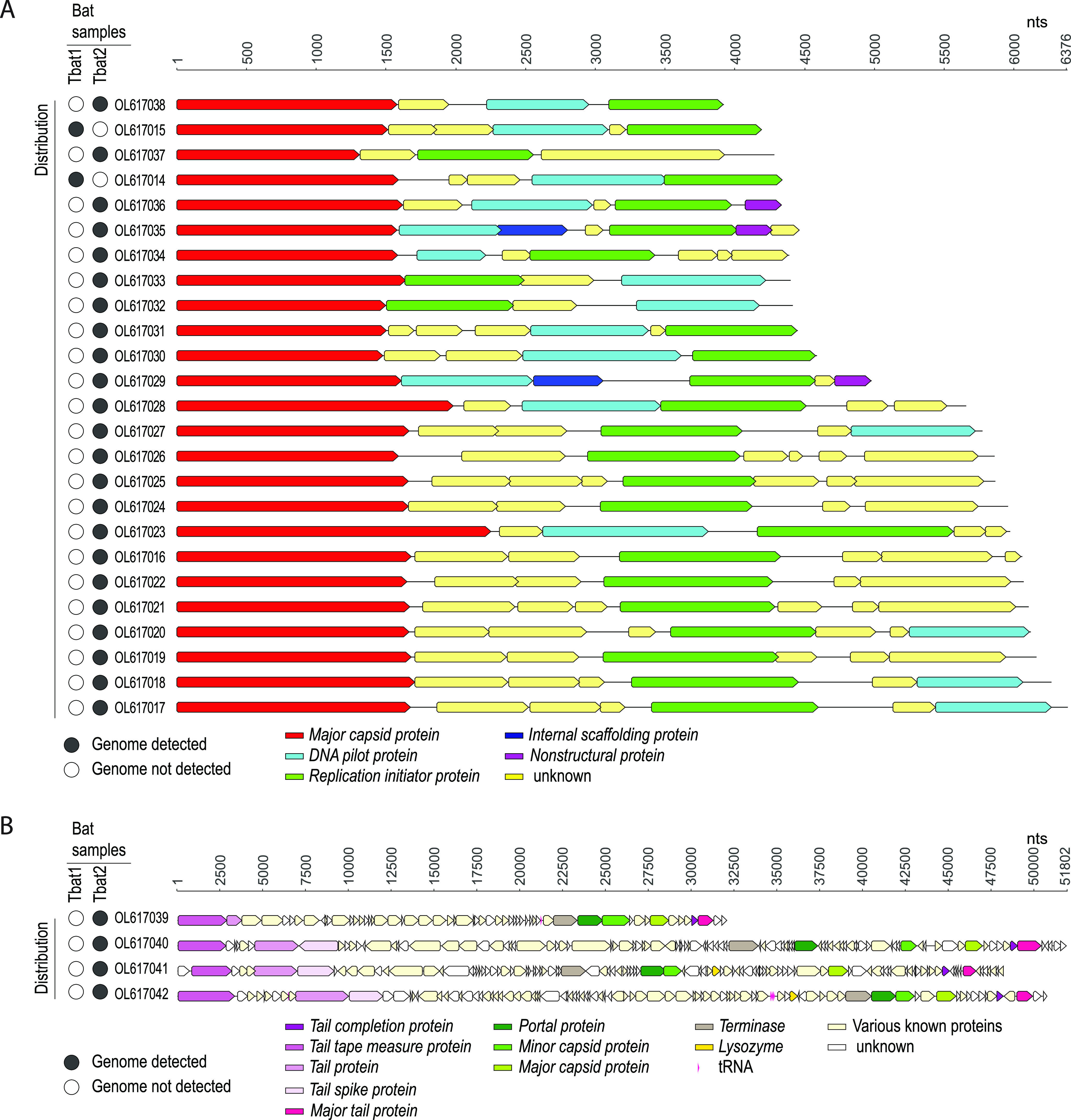
(A) Genome organization of the 25 microviruses from the Tongan bat fecal samples. (B) Genome organization of the 2 siphoviruses, 1 myovirus, and 1 podovirus from the Tongan bat fecal samples. Solid circles on the left indicate full genome coverage of the mapped reads in either the pooled sample of April 2014 or that of January 2015.

Of the 29 bacteriophages, 25 members of the *Microviridae* family (order *Petitvirales*) were identified in Tbat1 and 23 in Tbat2 (Fig. 1). These 25 microviruses have genome lengths of 3,911 to 6,376 nucleotides (nt) and GC contents of 36 to 60% (Fig. 1 and Table 1). All of the identified microvirus genomes encode a homologous major capsid protein and replication initiator protein, with the majority also encoding a recognizable DNA pilot protein (Fig. 1). BLASTn analysis against the nonredundant nucleotide database revealed that the microvirus genome with GenBank accession number OL617023 has the greatest nucleotide identity in this group, i.e., 86.8% (86% genome coverage) with respect to a microvirus identified in human samples (GenBank accession number BK033080) (13), whereas that with GenBank accession number OL617032 has the lowest nucleotide identity, i.e., 77.69% (2% genome coverage) with respect to a microvirus from soil (GenBank accession number KM589510) (14) (Table 1).

In the Tbat2 pooled sample, we identified four genomes in the order *Caudovirales*, i.e., two siphovirus genomes (48,082 to 50,667 nt, with GC contents of 47.7 to 48.5%), one myovirus (31,988 nt, with a GC content of ∼35%), and one podovirus (51,802 nt, with a GC content of ∼41%) (Fig. 1 and Table 1). Conserved tail and capsid protein coding regions were identified in these genomes (Fig. 1). The two siphoviruses share 72.15 and 94.03% nucleotide identity (70% and 24% genome coverage, respectively) with other siphoviruses from humans (GenBank accession numbers BK017157 and BK057309) (13). The myovirus (GenBank accession number OL617039) shares 82% nucleotide identity (4% genome coverage) with a genome from honeybees (GenBank accession number MN855801) (15), and the podovirus (GenBank accession number OL617040) shares 74.5% nucleotide identity (60% genome coverage) with a genome from Proteus mirabilis (GenBank accession number MN840487) available in GenBank (Table 1).

### Data availability.

The bacteriophage sequences have been deposited in the NCBI SRA under BioProject accession number PRJNA780525 (SRA accession numbers SRX13144068 and SRX13144069) and in GenBank under accession numbers OL617014 to OL617042.
